# Understanding COVID-19: A Hybrid Threat and Its Impact on Sport Mega-Events. A Focus on Japan and the Tokyo 2020 Olympic Games

**DOI:** 10.3389/fspor.2022.720591

**Published:** 2022-02-22

**Authors:** Solomon Imoudu Ilevbare, Gayle McPherson

**Affiliations:** School of Business and Creative Industries, University of the West of Scotland, Paisley, United Kingdom

**Keywords:** soft power, hybrid threat, mega events, Olympics, sport diplomacy, Tokyo 2020 Olympic games

## Abstract

The spread of the coronavirus (COVID-19) pandemic in 2020 affected the sports industry with the cancellation of many professional sports competitions worldwide. Thus, the postponement and organization of the Tokyo 2020 Olympic Games behind closed doors became a significant disruption to the global sports landscape. In this paper, we present a novel, conceptual discussion, signifying and defining the pandemic *via* the concept of international security as a hybrid threat. We associated the term hybrid threat to clarify better the difficult times facing sport mega-events. First, the paper proffers that the COVID-19 pandemic is a form of hybrid threat while reflecting on the connected implications of using sport as a soft power tool for nations. Secondly, we discuss the impact of COVID-19 on sport mega-events globally and explain the implications of COVID-19 on the Tokyo 2020 summer Olympic Games. This paper although drawing on some figures associated with COVID-19 and the Tokyo Olympic Games presents a theoretical contribution to knowledge in the area of sport mega events, soft power and hybrid threat. We outline how the threats, triggered by the pandemic, have impeded a successful Olympic Games and clarify how these threats have affected Japan's opportunity to use the Games as a soft power tool, which is the paper's key contribution to the field.

## Introduction

### What Is a Hybrid Threat?

This paper presents a conceptual discussion of the term hybrid threat and offers a re-conceptualization of the term to include COVID-19, going on to reveal how COVID-19 as a hybrid threat affected Japan's ability to use the Tokyo 2020 Olympic Games as a soft power tool to leverage the Games to their best advantage. We attest that COVID-19 as a hybrid threat, threatens all that the Olympics stands for, as a force for good, and suggest that COVID-19 as a hybrid threat to the Games, became symbolic of the hybrid threat to the world. We now discuss the contentious issues around this approach and tease out the different dimensions of hybrid threat in international security literature and propose a re-conceptualization of the term to include biological threats such as COVID-19, before revealing how this impacted, and will continue to impact, on sport mega-events.

The term hybrid threat identifies several forms of contemporary or twenty-first-century security issues to global security. It is the process of engaging in multiple forms of war simultaneously, with a dynamic combination of regular forces, irregular forces or criminal elements all unified to achieve mutually benefiting effects (Balaban and Mielniczek, 2018). Hybrid threat is characterized by the multiplicity of actors that blurs the traditional distinction between different types of armed conflicts and war (Hoffman, 2010; Wither, 2016). Nye (2009) defines hybrid threat as a threat where the method of conflict changes politically and economically, where non-military tactics and humanitarian efforts are involved; thus, utilizing a mixture of soft and smart power. In other words, hybrid threat involves a situation where a foreign power engages in activities to undermine the ability of another nation by exploiting domestic unrest instead of engaging in direct military actions. Therefore, it is difficult for the victimized government to pursue its internal objectives when subjected to hybrid threat, which consequently diminishes its role as a competitor globally. For example, the United States has continuously been a target of hybrid threat by states or non-state actors, such as cyber terrorism and extremist groups seeking to influence or disrupt US decision-making, society, and the political atmosphere (Balaban and Mielniczek, 2018). Wither (2016) points out that hybrid threat is defined by how states perceive and respond to hybrid threats, including which government agencies are involved in countering them. Hoffman (2010), in his adapted definition of hybrid threat from the National Defense Strategy, which is specific to the US, defines hybrid threat as:

“Any adversary that simultaneously and adaptively employs a fused mix of conventional weapons, irregular tactics, terrorism, and criminal behavior in the battlespace to obtain their political objectives (Hoffman, 2010, p. 3).”

His definition of hybrid threat was a development from the National Defense Strategy of 2005 and Quadrennial Defense Review (QDR) of 2006 that focuses hybrid threat on rogue states with nuclear weapons with likely elements of terrorism. Furthermore, Tromblay (2016) points out that hybrid threats or hostile activities can be categorized under four paradigms. The first is the nullification of political actors, where disagreements are created within a constituency to stop actors uniting around a policy. The second is the assistance to anti-government interest, which involves actors in society that are willing to attack rather than engage in the policymaking process with vitriol or violence. Third, is the creation of distrust in the government's policymaking process that saps its legitimacy. Lastly, it fills the needs or wants that the victimized government cannot thereby succeed in a specific area.

In addition, Hoffman (2009a,b) suggests that the characterization of hybrid threat under counterterrorism and unconventional warfare oversimplifies defense planning and resource allocation. However, Hoffman (2009a,b) bases his discussion on the re-conceptualization of hybrid threat, where he includes other forms of hybrid threats like irregular tactics, terrorism by non-state actors, and criminal behavior. However, the North Atlantic Treaty Organisation's (NATO) recent definition of hybrid threat now includes propaganda, deception, sabotage and other non-military tactics as agents of destabilization, and states they will defend the alliance and all allies against any threat, whether conventional or Hybrid (NATO, 2021). While this is a new and adapted definition of hybrid threat by NATO, the use of “other non-military tactics as agents of destabilization” makes the definition ambiguous. The definition of hybrid threat by NATO still subscribes to simply military tactics as a countermeasure to agents of destabilization. As a result, the definition lacks clarity on the method to combat non-military agents of destabilization, especially in identifying biological threats such as COVID-19 and more so when it is not a deliberate act by one agent or country against another. We have included it to show that a biological virus, although not a deliberate action, can impact a nation in the same way as if it was deliberate and therefore should be seen as a hybrid threat. Especially, if governments have to respond to it as if it was a hybrid threat.

The conceptualization of hybrid threat by the European Center of Excellence for Countering Hybrid Threats (Hybrid CoE) also highlights the lack of clarity and ambiguity in its definition of hybrid threat. For example, they point that hybrid threat refers to actions conducted by state or non-state actors, whose goal is to undermine or harm a target by influencing its decision-making at the local, regional, state, or institutional level, for example, in the political, economic, military, civil, or information domains (CoE, 2021).

Additionally, heads of state and government in Lisbon adopted the strategic concept of security in November 2010 which confirms NATO's commitment to deter and defend against any threat of aggression, and against emerging security challenges where they threaten the fundamental security of individual Allies or the Alliance as a whole (Aaronson et al., 2021). Aaronson et al. (2021) argue that The Strategic Concept, however, does not refer to hybrid threats or provide insight into the magnitude, likelihood, nature, or nuances of the emerging security challenges. Moreover, it does not address the possibility of having to face some or many of these challenges simultaneously, or the threat posed by the convergence of these many separate elements, which when braided together, constitute a threat of a different nature (Aaronson et al., 2021, pp. 111–113). They point out that the new threat confronting the diverse nations of the alliance is insidious and not easily defined or identified. It flourishes in layers between states and in the soft areas of bad or weak governance.

In addition, they suggest that the new threat consists of distinct but tangled elements—hence the title hybrid. This is because of the interrelatedness of their constituent elements. The complicated and interdependent nature of the activities required to counter them, the multiplicity of key stakeholders with vested interests, and the dynamic international security environment in which traditional military solutions may not be best (or even a key component) but may nevertheless be necessary (Aaronson et al., 2021, pp. 111–114). Jasper and Moreland (2014) point out that the definition of hybrid threats is implicitly related to the globally de-stabilizing effects of the post-Cold War era that created conditions for their development, coupled with the rapid appearance of disruptive technologies and mass communications media shortly thereafter that offered these nascent groups expansive propaganda networks and novel military tools. However, Jasper and Moreland (2014) went on to adopt and identify six revised characteristics of hybrid threat from Hoffman's (2010) definition of the term to include blended tactics, flexibility and adaptable structure, terrorism, adversaries, propaganda and information warfare, criminal activity and disregard for international law. To clarify our intended argument on identifying COVID-19 as a hybrid threat, we have set out below a table identifying the key definitions of hybrid threat and similarities between them before then making a case for the need to include COVID-19 as a hybrid threat. In doing so, we then suggest a new definition or re-conceptualization of hybrid threat as it relates to the Tokyo 2020 Olympic Games derived from this evidence.

### Definitions and Similarities of Hybrid Threat

It is useful to review some of the definitions and the similarities between key authors to reveal and demonstrate how this informed our re-conceptualization of the term hybrid threat and add to the conceptual understanding of the term.

Aaronson et al. (2021) suggest that “Hybrid threats are those posed by adversaries, with the ability to simultaneously employ conventional and non-conventional means adaptively in pursuit of their objectives” (p. 115).

Jasper and Moreland (2014) assert that although there is not a universal definition for a hybrid threat, NATO uses the term to describe “adversaries with the ability to simultaneously employ conventional and non-conventional means adaptively in pursuit of their objectives” (p. 4). For example, the US Army's Special Operations Command advocates for “hybrid organizations and structures, which combine the mission command for special operations and conventional forces” (p. 6). They go on to say that:

“supporters of a hybrid threat concept counter that contemporary threat actors are creating a new type of warfare through the employment of 21st-century technologies and communications networks, unrestricted operational art, and novel combinations of conventional and non-conventional capabilities that are distinct from traditional irregular warfare methods” (p. 2).

Jasper and Moreland (2014) suggested the following six revised hybrid threat characteristics are offered to provide further clarity:

**Blended Tactics**. Hybrid threats combine conventional military capabilities with small unit guerrilla tactics, asymmetric attacks, and highly mobile standoff engagement systems.
**Flexible and adaptable structure**. Hybrid threats are generally composed of paramilitary forces that can organize both in massed conventional formations and as small, distributed cells. Hybrid threats create a governance component to establish stability and sustain operations.
**Terrorism**. Hybrid threats utilize terror campaigns to proliferate hate and despair and to strike fear in adversaries. They target cultural icons and symbols to destroy the identities, heritages, and belief systems that oppose their ideologies.
**Propaganda and information warfare**. Hybrid threats exploit global communications networks to spread extremist views, raise funds, and recruit.
**Criminal activity**. Hybrid threats use crime and fundraising as reliable sources of revenue to fight, train, recruit, govern, and sustain operations.
**Disregard for International Law**. Hybrid threats cynically view international laws as a constraint upon their adversaries that can be exploited.

Hoffman (2009a,b) was among the first to propose clear hybrid threat characteristics that might be meaningful and useful to planners:

**Blended modalities**. Hybrid threats use a combination of conventional and non-conventional tactics combined with terrorism and criminal activities.
**Simultaneity**. Hybrid adversaries can employ different modes of conflict simultaneously in a coherent way.
**Fusion**. Hybrid threats are comprised of a mix of professional soldiers, terrorists, guerrilla fighters, and criminal thugs.
**Criminality**. Hybrid threats use criminal activity to sustain operations and, in some cases, as a deliberate mode of conflict

And from this provided the overall definition:

“Any adversary that simultaneously and adaptively employs a fused mix of conventional weapons, irregular tactics, terrorism, and criminal behavior in the battlespace to obtain their political objectives” (Hoffman, 2010, p. 3).

The European Center for Excellence on Countering Hybrid threats definition is particularly revealing suggesting that:

“The term hybrid threat refers to action conducted by state or non-state actors, whose goal is to undermine or harm a target by influencing its decision-making at the local, regional, state or institutional level. Such actions are coordinated and synchronized and deliberately target democratic states' and institutions' vulnerabilities. Activities can take place, for example, in the political, economic, military, civil or information domains. These activities are conducted using a wide range of means and designed to remain below the threshold of detection and attribution” (CoE, 2021, para. 1).

And they go on to discuss hybrid action in relation to the blur between actions:

“Hybrid action is characterized by ambiguity as hybrid actors blur the usual borders of international politics and operate in the interfaces between external and internal, legal and illegal, and peace and war. The ambiguity is created by combining conventional and unconventional means – disinformation and interference in political debate or elections, critical infrastructure disturbances or attacks, cyber operations, different forms of criminal activities and, finally, an asymmetric use of military means and warfare” (CoE, 2021, para. 2).

Lastly, The North Atlantic Treaty Organization (NATO) recent definition of hybrid threat now includes:

“Propaganda, deception, sabotage, and other non-military tactics as agents of destabilization, and to defend the alliance and all allies against any threat, whether conventional or Hybrid” (para. 1).

We have reviewed the above key authors' definitions and examined them for similarities to inform our re-conceptualization of hybrid threat. We found that from the key actors above the similarities in definitions of hybrid threat includes:

Adversaries
Ability to simultaneously employ conventional and non-conventional means adaptively
Special operations and conventional forces
Conventional and non-conventional capabilities that are distinct from traditional irregular warfare methods.
Conventional military capabilities with small unit guerrilla tactics, asymmetric attacks.
Utilize terror campaigns to proliferate hate and despair.

In the next section, we move on to discuss how this links with more traditional definitions of threats and security and present our definition of hybrid threat.

### Addressing the Traditional Definition of Threats and Security

The definitions mentioned above have identified the similarities in the definition of hybrid threat, however, their definitions of hybrid threat subscribe to only military expertise because of the recurring characteristics of hybrid threat that include conventional and non-conventional capabilities that are distinct from traditional warfare. And the multiplicity of key stakeholders with vested interests, including the dynamic international security environment, provides a probability that the traditional military solutions may not be the best option (or even a key component) and may not be necessary for countering the emergence of the new hybrid threats. This consideration alone points to a need to revisit the definitions and practices on the fight against hybrid threat. The faster we understand these impacts, the better it will be to address them. The socio-economic and security effect of COVID-19 confirms that associating the term hybrid threat with the pandemic should not be discounted. However, while we seek to justify COVID-19 as a hybrid threat, we distinguish that COVID-19 is a hybrid threat with no aggressor, such as a state or non-state actor. This means that COVID-19 is a biological threat that has exploited the vulnerabilities of states' institutions globally but not a biological weapon used by a state or non-state actor to exploit states' vulnerabilities. Furthermore, the reports from the investigation made by the WHO and the US about the origin of the virus were inconclusive. Still, they dismissed the possibility that the virus might have leaked accidentally from the Wuhan Institute of Virology or been utilized as a biological weapon by a state or non-state actor (BBC, 2020a,b, 2021a,b). However, irrespective of the controversial or inconclusive reports on the origin of COVID-19, human beings have become the agents of COVID-19 used by the virus to spread infections. We have re-conceptualized the definition of hybrid threat from the above definitions, to suggest:

“Hybrid threat refers to actions conducted by state, non-state actors, including the emergence of biological human threats or invaders (Viruses), whose goal is to undermine or harm a target at the local, regional, state or institutional level but not necessarily planned in the case of a virus. Such actions may be coordinated and generated by artificial and biological agents to synchronize and target the vulnerabilities of democratic states. This includes propaganda, deception, sabotage and other non-military tactics, including the emergence of biological threats like COVID-19 as agents of destabilisation.”

COVID-19 revealed the lack of preparedness from nations and states and exploited the vulnerabilities of the social, political and economic institutions that govern the day-to-day running of the society. Therefore, the emergence of the pandemic allows for a rethink to identify COVID-19 as a hybrid threat. Thus, we depict the need for a re-conceptualization such as ours above of the definition of hybrid threat to include biological threats such as SARS, Rika virus, Ebola and now COVID-19 that may lead to pandemics. Irrespective of the controversial or inconclusive reports on the origin of COVID-19 that led to its spread and infection, we suggest that other agents of the COVID-19 pandemic, which made the virus, are misinformation, conspiracy theories on the emergence of the pandemic, and at the same time, the uncovered social reality of poverty and social inadequacy, insecurity and inequality from government institutions leading to a lack of trust from the public. These agents were instrumental in the initial difficulty to counter the spread of the virus. Uniquely, we now address this issue further by drawing the relationship between hybrid threat and sports mega-events, discussing as an illustrative example the effect of COVID-19 in mega sports-events like the Tokyo 2020 Olympic Games as a form of hybrid threat (TBA, 2021). The key phrases from the definitions are “target democratic states' and institutions”' “vulnerability.” Therefore, the impact of COVID-19 on state institutions revealed the lack of preparedness and exploited the vulnerabilities of the social, political and economic institutions that govern the day-to-day running of society. Therefore, the emergence of the pandemic allows for a rethink to identify COVID-19 as a hybrid threat. Uniquely, we seek to address this issue further by presenting a conceptual analysis, drawing a relationship between hybrid threats and sports mega-events, discussing as an illustrative example the effect of COVID-19 in mega sports events like the Tokyo 2020 Olympic Games as a form of hybrid threat.

### Hybrid Threat and Its Association to Sport Mega-Events

From the above definitions, terrorism has been the most common form of hybrid threat to state governments and society. The definition of terrorism or conventional or irregular warfare is ambiguous and not universally accepted because of the different modes of attacks against civilians, non-combatants and the evolving trends of terrorist groups and their objectives. Scholars such as Butko (2006), Sinai (2008), and Shanahan (2016) have all discussed the definitions, methodological issues and conceptual issues of terrorism. Their definitions of terrorism have common characteristics such as violence, harm, threat, and politically or religiously motivated attacks against civilians. However, we recognize these common characteristics as hybrid threats because the evolving tactics used by terrorists targeted toward civilians makes the attack hard to combat or detect quickly. However, we are not trying to delve deep into the critique and definitions of terrorism as a hybrid threat but instead to discuss conceptually COVID-19 as a hybrid threat and its effect on the Tokyo 2020 Olympic Games as a novel paradigm shift. This means that we place COVID-19 under the same umbrella as terrorism as a hybrid threat but with different origins and modes of attacks. To justify this narrative, the delivery of sport mega-events post 9/11 saw a sharp increase in the security budgets of sport mega-events. For example, the 1992 Barcelona Olympics security budget was US$66.2 million, by the 2008 Beijing Olympic Games, this had risen to US$6.5 billion (Giulianotti and Klauser, 2012). Because of the large spectator participation nature of sport mega-events, cities witness an influx of people attending sport mega-events like the Olympic Games. The management and organization of such events includes security measures, counterterrorism measures like CCTV surveillance, the presence of police, and counterterrorism police officers. In the organization of sport mega-events, the perceived risk of terrorism has been argued to deter spectators from attending sport mega-events (Cashman, 2004). Solberg and Preuss (2005) state that tourists who regularly attend sport mega-events are increasingly likely to avoid such events because of terrorism concerns. For example, the 2004 Athens Olympic Games recorded low crowd attendance, which was attributed to fears of terrorism (Toohey and Toohey, 2007). Furthermore, Taylor and Toohey (2003) point out that the gain for terrorist organizations in targeting sport mega-events like the Olympic Games or World Cup Football is high as it affords public visibility, global media exposure, and the symbolic representation of these events. Thus, there is a need to assess the risk management of terrorism in organizing sport mega-events. Furthermore, Schimmel (2006) points out that the increased militarisation of mega sports events and urban spaces has been a landscape where military tactics are necessary to protect capital investment, enable crowd control, as well as countering or preventing terrorism. However, all these measures fit neatly within the paradigm of traditional security threats. Therefore, COVID-19 as a hybrid threat shares the same similarities with terrorism on sport mega-events. This is because of their effect in limiting the attendance of tourists and sports fans from attending sports competitions.

### The Interplay Between Sport Mega-Events and Hybrid Threat

In examining the interplay of hybrid threat in the context of sport mega-events, it is important to give historical and contemporary accounts of sport mega-events that have been affected by hybrid threats such as terrorism. For example, in the 1972 Olympics Games in Munich, the Palestinian terrorist group Black September attacked the Olympic Village where the Israeli team were housed, leading to the death of 11 athletes and 1 German Police Officer. In Atlanta, the 1996 Olympic Games was subjected to a pipe bomb exploding in the continental Olympic Park (Sky News, 2017). In 2015, three suicide bombers blew themselves outside of the Stade de France in Saint-Denis in Paris, where France and Germany were playing an international friendly football match, and the Islamic State claimed responsibility for the attack. In the case of the 2012 London Olympics, the bidding team for London had to reveal its resilience to anti-terrorism before the International Olympic Committee and an international audience to gain national support for the proposed security budget on power surveillance and social control to help against terror attacks (Giulianotti and Klauser, 2012). The above cases show the interplay between sport mega-event and hybrid threat such as terrorism, confirming the reason for the increasing militarisation of sport mega-events. As a result, we argue from the above discussions that the interplay of hybrid threats or adversaries to sports mega-events is not new or unfamiliar in sports management.

We also argue that, because our re-conceptualization of hybrid threats can impact sports mega-events, despite countries including in their bid the securitisation processes of the sport mega-event in question; they have never had to plan for hybrid threats previously. The traditional definition of hybrid threats in the international political system has spilled over to the organization of sports mega-events, leading to the militarisation of sport mega-events (Giulianotti and Klauser, 2012). It is also important to note that it is easy to associate the term “militarization” with sport mega-event because governments are heavily involved in organizing sport mega-events to boost their political and diplomatic ideology (Grix and Lee, 2013; He et al., 2020). Therefore, it leads to the transfer of state political and security methods and practices into sport mega-events. However, we contest that the continuous definition of the driving forces of conflict or threat exclusively to military tactics and counterterrorism measures that include predicting state behavior in global politics needs to be refined. However, there might be relevant discussions and debates why the definitions of hybrid threat should not include biological threats, but the evidence of the pandemic shows otherwise. This is because the emergence of COVID-19, leading to a pandemic, has targeted and exploited the vulnerabilities in state institutions. We are suggesting that people should not identify COVID-19 as a military adversary or weapon used by state and non-state actors but instead, we have mentioned above how the hybridity of COVID-19 and its impairment on the international and national economy, health and social security has affected all nations. Specifically, its impact on sport mega-events like the recently concluded Tokyo 2020 Olympic Games and the concomitant knock-on effects of soft power relations afforded to the nations hosting sport mega-events.

### The Importance of Hosting the Tokyo 2020 Olympics as a Sport Mega-Event

As we assert COVID-19 as a hybrid threat to sport mega-events, specifically in the case of the Tokyo 2020 Olympic Games, this section is important because it shows the significance of hosting sports mega-events like the Tokyo 2020 Olympic Games. Flyvbjerg et al. (2020) reveal that the Tokyo Olympic Games cost US$15.4 billion, the most ever, but this is likely because of increased over-run costs due to the delay of the Games (Flyvbjerg et al., 2020). Normally, the large influx of tourism means a massive influx of tourism dollars, which is part of the overall impact of hosting the Olympic Games, but Tokyo did not benefit from that in 2021. Increased benefits also include publicity and international exposure of the host city to benefit global trade and capital flow (Billings and Holladay, 2012). Most countries have used sport mega-events like the Olympic Games and FIFA World Cup as a vehicle for bringing forward planned developments they wanted to make, so the costs revealed are not necessarily solely Games-related (Flyvbjerg et al., 2020). The Games then are being used as a tool for soft power and public engagement to enhance a nation's policy objectives, cultural relations, increase tourism, national pride and nation branding, the five key dimensions to which Grix et al. (2020) and Grix and Lee (2013) referred. In Japan's case, the Tokyo 2020 Olympic Games may have become a tool for increased tourism and a showcase of national pride; highlighting their role as the peaceful nation achieved with the 1964 Games. However, it may be difficult to measure the extent to which this was possible because of the organization of the Games behind closed doors with no presence of fans and sports tourists. Prior to the pandemic, Japan was able to showcase its culture such as manga, anime, and games (MAG) which is part of the main source of Japan's soft power strategy “Cool Japan.” For example, when the Olympic flag was passed to Japan during the 2016 Rio Olympics, Prime Minister Shinzo Abe dressed as a Super Mario and supported by athletes shown in a video dressed like Hello Kitty and Doraemon (Rich, 2016). But as suggested, it was difficult to measure the importance of Japan's soft power strategy at the time of the Games, especially, because of how the Games were held. Using the games as a soft power tool they expected to create global visibility, presenting Japan's image and identity to the world. Japan had fallen recently in the global soft power rankings, from 5th in 2018 to 7th in 2019 and Japan sought to use the Games to catapult itself back up the rankings (Portland, 2019; McPherson and Ilevbare, 2021). The IOC worked with Japan and other nations to present a “stronger together” narrative of using the Games to unite people digitally due to the COVID-19 pandemic encouraging people to get physically active and help their mental resilience alongside athletes making their journey to the Games (IOC, 2021) but that was fraught with difficulties given athletes themselves were catching COVID-19. The Games may have become a tool for public diplomacy with the presence of the global media and broadcasting of the Games to the foreign public. However, the impact of the media on Japan's soft power was limited right up until the Games with negative stories in the press regarding sexism etc; doing little to help their soft power advantage (McPherson and Ilevbare, 2021).

Horne (2007) points out that the growing attraction of sport mega-events in soft power terms has been for three main reasons. First, is that the development of new mass communication technologies, including satellites, provides hosts with an increased global reach from events like the Olympic Games and FIFA World Cup. Second, the influx of serious corporate sponsorship money has provided an essential and alternative source of income for the sponsors and host cities. The third is that countries host sports mega-events as a useful tool in selling both commercial products and brand awareness of their country as a destination for investment; which both ultimately lead to a higher placing in the soft power rankings. As McPherson et al. (2017) attest sport mega-events present countries with the potential to use them as a vehicle for progressive opportunity to make a change and promote themselves, displaying their allures to global audiences, attracting tourism, outside investment and cultural exchange, which are key tenets of soft power relations (Horne, 2007; Grix and Lee, 2013). From the lens of Joseph Nye's concept of soft power, Grix and Houlihan (2014) discusses sport mega-events as part of a nation's soft power. His investigation on soft power is that countries use sport mega-events to alter their image among the foreign public. For example, Germany went through a long and well-planned process of transforming its image by hosting the 2006 FIFA World Cup (Grix, 2013). In the USA, the move to host sport mega-events was a response to the federal government's reductionist approach to economic development funding, so the neo-liberal reaction from the federal government was to develop a strategy that could see urban economic growth in the 1980s (Foley et al., 2011). Since the success of what has been deemed the first commercial Games of Los Angeles, in California in 1984, the federal government has realized the capacity for using mega sports events for soft power purposes as well as urban developmental and growth strategies, which developing nations are keen to pursue (Grix and Lee, 2013). Furthermore, the use of mega sports-events as a soft power tool demonstrates the growing interest in the relationship between sport and politics and the leveraging of sport mega-events by most countries, especially in the case of emerging power countries in recent years (Foley et al., 2011; Rowe, 2018). Emerging power states such as Brazil, Russia, India, China and South Africa, known as the BRICS nations, have all hosted sports mega-events as a tool to reach out to the foreign public in other to boost their soft power. Sport and diplomacy have been contested as Games within Games since the mid-2000s, and (Rofe, 2018) elaborates on the interplay of sport as a diplomatic tool in his book “Sport diplomacy, Games within Games.” In addition, it is important to note that sports mega-events, once seen as a relatively cheap medium for governments of all kinds of political ideals to improve their soft power, have become very expensive to host, as demonstrated above (Grix et al., 2020). Sport mega-events require that a large sum of money is paid for the privilege of hosting and organizing the event. This exclusive policy has undoubtedly put some host nations out of the frame (McGillivary and Turner, 2018).

### The Broad Case of Japan and the Motivations of Hosting the Tokyo 2020 Olympic Games

In Japan's case, the 1964 Tokyo Olympic Games was a significant cultural achievement for Japan as a technology leader. This achievement included displaying new technology during the Games, like introducing a new timing device for swimming, introducing the transistor radio and camera that became popular in the 1950s and 1960s (IOC, 2020; Droubie, 2022). In addition, the 1964 Tokyo Games was the first Olympic Games to be aired on color TV across the globe through satellite, a joint Japan and US project, which was good for Japan's interaction with the foreign public and brand (Collins, 2011; Abel, 2012). However, Japan hosted the Winter Olympic Games in 1998 and the Korea/Japan FIFA World Cup in 2002. While the 1998 Winter Olympic Games is classified as an international sports event, it is not as symbolic as the global visibility of the 1964 Summer Olympic Games in Tokyo. The 1964 summer Olympic Games holds more significance in Japan's imperial, cultural and war history into a peaceful nation and its reacceptance into the global community after the Second World War (Horton and Saunders, 2011; IOC, 2020). Furthermore, aside from the historical and symbolic significance of the Tokyo 1964 Olympic Games, the motivation and importance of the Tokyo 2020 Olympic Games can be identified through the lens of Japan's former Prime Minister Abe's political ambition to revise Article 9 of Japan's constitution to allow a shift from pacifism to a more proactive role for the military (Kaufman, 2008; Easley, 2017). For example, he wanted to develop more hard power weapons and at the same time employing soft power strategies to demonstrate to others he was aiming to become a top tier country in power terms and part of his strategy was to show he had a significant role in the bid to host the Tokyo 2020 Olympic Games. Abe in 2012 aimed to strengthen Japan's position domestically, regionally and internationally as a first-tier country. His proposed constitutional amendment or review and re-interpretation of Japan's article nine to allow for a more proactive defense military was one phase of Abe's ambition to make Japan a significant player in the international community (Huges, 2006; Basu, 2017). For the Abe government, the priority was to change Japan's pacifist constitution into a proactive military defense (Oros, 2015). A hard power strategy, but for his hard power strategy to work effectively, hosting the Tokyo 2020 Olympic Games as a soft power resource became a strategy to improve Japan's foreign policy and image. The Tokyo 2020 Olympics Games became a soft power strategy to enhance Japan's national image, building upon the hosting of the Rugby World Cup the year before, these were seen as the most important events since hosting the 1964 Tokyo Olympics. Fast forward to more than 55 years and Japan has staged the Tokyo 2020 Olympic Games, five decades since hosting its first summer Olympic Games. The Games have changed, including how the Olympics Games are viewed, the highly competitive nature of emerging power states bidding to stage the Games, and the Games becoming an opportunity for emerging states to improve their image and credibility in global affairs (Cornelissen, 2010). In addition, viewing through the lens of Asian Geopolitics and the use of the Olympic Games as a tool for nation branding, the 2008 Olympic Games had a global and national significance for Beijing and China. In 2008, in a closing ceremony speech by the President of the International Olympic Committee, Jacques Rogge, stated that the event had advanced international understanding between the hosts and other societies: “Through these Games, the world learned more about China, and China learned more about the world” (Giulianotti, 2015, p. 4). Giulianotti (2015) discussed the 2008 Beijing Olympics as a soft power tool and disempowerment, referencing Zhongying (2008), a Chinese international relations expert. The Beijing Olympics was a breakthrough in the exploration of China's soft power. For example, while providing a unique occasion that drew the outside world's attention to the event and China as a host, the Games helped embed China fully within international society (Giulianotti, 2015). We can see this from the turnaround of the world media to report positively on the changes Beijing had made to air pollution, opening up media access beyond their internal social media platform, “weibo,” to other platforms during the Games, as well as addressing the issue of human rights abuse, all of which saw China rise in soft power terms (Manzenreiter, 2010). In addition, the Olympic Games helped China strengthen its link with competing nations, such as the United States and developing countries. In relation to using the Games as a soft power tool, Japan is at the heels of China, considering China's position in global affairs and East Asia, especially after hosting the 2008 Games that created global visibility and with China gearing up to host the 2022 winter Olympics. Outside of internationalized cities like Tokyo and Osaka, Japan is one of the least diverse countries globally, known for its cultural homogeneity (Burgess, 2010). This is a significant drawback for Japan, and for young Japanese, the older generation's outlook remains frustratingly arcane, their traditional outlook lamented as an obstacle to social progress. There is an aging population in Japan as nearly 30 per cent of the country's 126 million people are over 65 (Tsuya, 2014, Forum 005 Special Report), the Tokyo 2020 Olympic was an opportunity to look toward a more outward-looking and inclusive future. This aspiration? explains the Games organisers' vision to put on a show that would inspire and champion the next generation. In addition, Tokyo is a city with more than 37 million people. It is technologically advanced, culturally rich for tourists with an abundance of Japanese iconography, the mix of the old and new, as well as anime stores, manga and giant Pokémon (Okuno, 2014; Kartikasari, 2018). Tokyo was set to be Japan's best social influencer through which the world will connect to Japanese culture if only they had the opportunity to showcase their culture fully to an international audience. The just-concluded Tokyo 2020 Olympic Games may have opened the door for Japan to exercise its agency, political and social ideals on the international stage. However, with the emergence of COVID-19 and its disruption to the organization of the Tokyo 2020 Olympic Games, it is plain to see the impact of COVID-19 as a hybrid threat on the 2020 Tokyo Olympics and Japan's soft power. For example, the Tokyo 2020 Olympics did not meet its expectations in terms of global visibility and interactions with the foreign public. Research has shown that the Games recorded a fall in television audiences in comparison with the 2016 Olympics (Coster, 2021). The Games became the least-watched across Europe and America, recording the smallest audience in the past 33 years (Coster, 2021). This example points to the fact that COVID-19 is a hybrid threat, and in Japan's case, it has impacted Japan in the area of tourism, global visibility or interaction with the foreign public, economically and in some cases, low public acceptance both nationally and internationally. Therefore, the impact of COVID-19 may well have depleted Japan's financial and political calculation of using the Games as a soft power tool. In conclusion, the impact of COVID-19 on Japan and its hosting of the Games signals that COVID-19 is a hybrid threat given the many fronts on which Japan has been negatively impacted.

### The Implications of COVID-19 as a Hybrid Threat to Tokyo 2020 Olympic Games and Japan's Soft Power Advantage

The emergence of the COVID-19 pandemic now identified as a hybrid threat means several factors may have altered Japan's ability to use the Olympic Games as a tool to enhance its image. In Olympic history, the outbreak of diseases has always been a manageable, albeit dangerous, risk. For example, the Salt Lake Winter Olympics experienced a flu outbreak; during the Vancouver Winter Games, measles and the case of the norovirus in Pyeongchang. Unlike flu and measles, COVID-19 brings a different kind of threat resulting in a pandemic; one that no nation had encountered previously. Previous studies have shown that scientists have predicted a pandemic's frequency and possibility; for example, the Zika virus was an adversary that almost disrupted the 2016 Rio Games, not forgetting the SARS outbreak in the 2004 Athens Games. With the emergence of COVID-19, one would think that there were adequate measures and Plan B for mega-events like the Olympics because of the complicated procedures and steps countries have to go through to host the Games. The lack of Plan B against the emergence of a disease or pandemic is a dent and complex problem that the IOC and other sport mega-event organizers must solve. With the pandemic, the centralization of a global event, especially the Olympic Games, has not proven to be effective economically and socially and reduced Japan's ability to achieve the goals it had set itself in securing the right to host the event. This is because of the IOC and the Japanese government's inability to accommodate sports fans and tourists to Tokyo because of the fear of a spike in COVID Infections. Organizing an international event like the Olympics in a single geographical location like Tokyo has resulted in questions regarding even distribution of wealth, legacy and income in the past (McBride, 2018). The emergence of COVID-19 has exposed the vulnerability of the Games as a product with no risk management strategy identified from event owners such as the IOC. The vulnerability of the Games questions the Games economic, political and athletic viability in their current state (Minter, 2020). This vulnerability was demonstrated with the emergence of the COVID-19 pandemic that exploited the vulnerabilities of the Tokyo 2020 Olympic Games in terms of the inadequacy of the Games organizers to prevent hosting the Olympics behind closed doors, which in turn affected the economic, political and athletic plan of the event.

**Figure 1:**
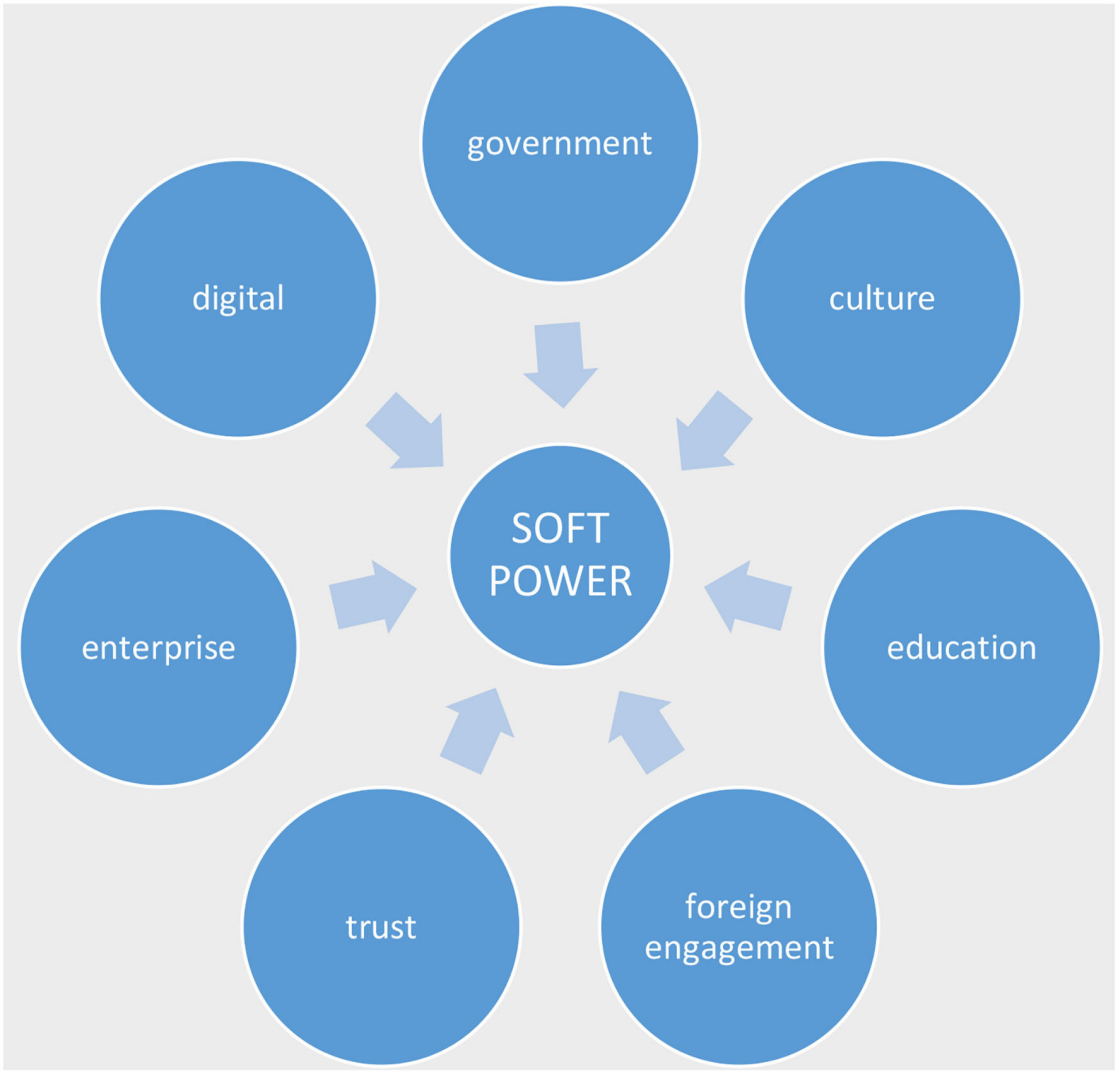
Categories of power.

Since we assert that sports mega-events and the Tokyo Games can act as a soft power tool for Japan, we place the categories of culture, digital, foreign policy, engagement, and interaction with the foreign public under the umbrella of soft power. Soft power is recognized as shaping the preferences of other people in terms of the appeal and attraction of a city or country to them, which is consistent with characteristics of soft power such as culture, political values and foreign policies (Nye, 2009). To Nye (2009), the benefit of engaging in soft power practices is legitimacy, credibility, and efficacy. In the case of Japan, to understand the importance of the Tokyo Olympic Games as a soft power tool and to reveal how it has been affected by COVID-19, it is important to outline the categories of soft power that the Olympic Games fall under, revealing its importance as a soft power tool for Japan. Figure 1 explains the categories of soft power such as culture, digital, foreign policy, engagement, and interaction with the foreign public. We are also mindful of the global indices that test categories of trust and quality of life in countries, so we have added these below. These categories are essential for a country like Japan seeking to gain more influence, attraction and legitimacy. Normally, the soft power influence is judged from an external audience, but it is important to have the internal approval of citizens as they influence external markets through digital media. This paper uses this model of soft power as a conceptual framework to determine the effectiveness of Japan's soft power strategy using the 2020 Tokyo Olympics. In linking the categories of soft power to a sport mega-event, the paper theorizes how Japan could have used the Tokyo Olympic Games as a soft power vehicle albeit curtailed somewhat with the hybrid threat of COVID-19.

**Figure 2:**
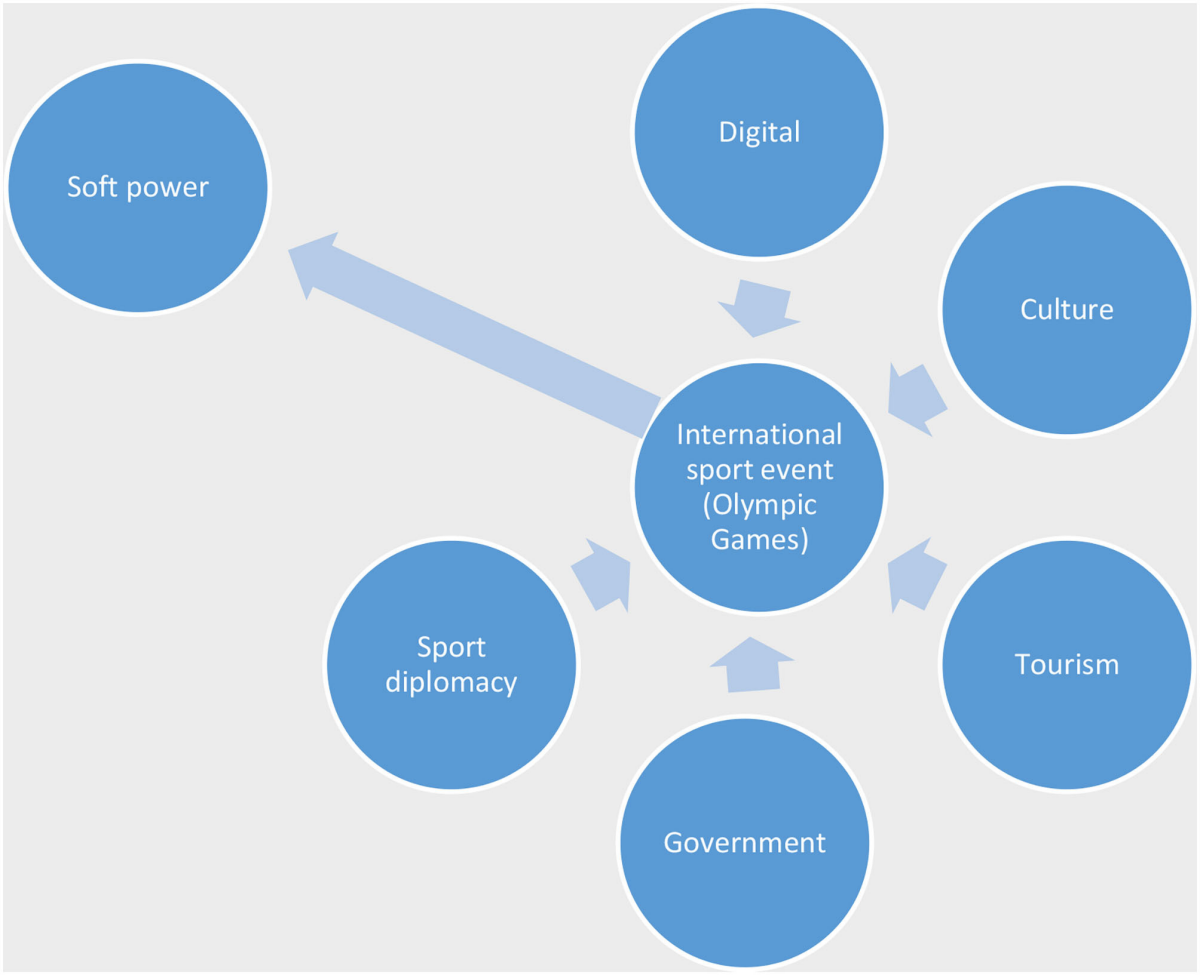
Component of soft power and international sport event.

Furthermore, Figure 2 clarifies that international sports competitions such as the Olympic Games are an important vehicle for soft power. The figure shows the categories of international sports competitions: digital, culture, tourism, sports diplomacy, and government. This means bringing all categories of soft power practiced separately and probably at different times by different government ministries together by hosting the Olympics. Therefore, hosting the Tokyo 2020 Olympic Games could have served as a significant advantage in promoting its soft power whilst giving the benefits these categories would bring to Japan's international image had they been able to exploit the event properly.

In this case, sport mega-events such as the Olympic Games fall under the umbrella of soft power. This means that the Olympic Games conveys all categories of soft power such as digital, culture, foreign engagement at the same time while connecting Japan to the globe through sport. However, it was difficult for Japan to use the Games as the soft power tool they had hoped for due to the insurgence (not deliberate) of COVID-19. The 2020 Tokyo Olympic Games were held behind closed doors with no sports fans and mega-event tourists traveling to Japan. However, organizing the Games without sports fans and mega-event tourist defeats some of the purposes of using the Games as a soft power tool. However, we point out that the emergence of COVID-19 has impacted three categories in using the Games as a soft power tool. The first is on public diplomacy, secondly on cultural diplomacy and thirdly on economic growth. Grix and Lee (2013) point that the hosting of sport mega-events provides emerging states with the opportunity to practice public diplomacy. They suggest that hosting sport mega-events is a political practice. In the past few years, countries with unattractive political ideals or a history of human rights issues or abuse have all hosted the Olympic Games or sport mega-events to gain a new positive image, attraction, or change their already existing stereotype (Lucy, 2017). In Japan's case, hosting the Tokyo 2020 Olympic Games with no fans and no external tourism has limited public diplomacy practice. This is one of the detrimental effects of COVID-19 as a hybrid threat on the Tokyo 2020 Olympic Games as a soft power tool for Japan. For example, watching the games from the comfort of our homes defeats the purpose of physical attraction to culture and projects? an incomplete media representation of Japan's image. Secondly, sport mega-events are often the instrument of cultural policies. Sports mega-events create opportunities for the host country to make a significant cultural statement to promote its image internally and attract visitors (Getz, 2012; Pop et al., 2016). Bond et al. (2007) point out that culture plays a vital role in international relations from connective human values. Culture is a means for people to understand themselves, primarily through cultural exchanges where people can appreciate commonality and differences (Bond et al., 2007). Hosting sport mega-events while portraying a positive and attractive image provides the host country with the opportunity for economic development, increased tourism, and in some cases, the return of huge investments. The Olympic Games provide a platform for identity politics where international and domestic cultural exchanges create platforms for negotiations and finding common values. It provides a safe platform for unofficial political dialogues and building relationships between political actors, including opening negotiations between hosts and other countries, especially difficult political relationships (McGillivray and McPherson, 2012; Giulianotti, 2015; Brannagan and Giulianotti, 2018). A country's image holds capital value when attached to the sport mega-event narrative. These reasons, among others, are motives for the competitive bid to host a sport mega-event. Before hosting the Tokyo 2020 Olympic Games, Japan has engaged in several cultural and international exchange programs domestically and overseas. Japan's Prime Minister appointed the first minister in charge of the Cool Japan strategy. The strategy aims to share Japan's unique food, fashion, and traditional culture with the rest of the world while highlighting Japan's hospitable culture (Daliot-Bul, 2009). By promoting these cultural values, Japan believed that its economy could be revived, encouraging tourism and strengthening diplomatic ties abroad. However, Cool Japan is sometimes criticized for mainly pleasing people interested in Japan's subcultures and arguing that the subcultures they base their communication on are not seen as positive in Japan (Valaskivi, 2013). Therefore, hosting the Olympic Games as a strategic tool for its soft power was an opportunity and a cultural hub to foster Japan's cultural ambitions. The uncertainty caused by the pandemic became a major problem for Japan; the Tokyo 2020 Olympic Games may have become an opportunity to display Japan's unique cultures and ideas behind? the Cool Japan strategy, but this goal has not been achieved. However, we will not know the impact of hosting a closed mega-event like the Tokyo 2020 Olympics for several years. Finally, the perceived economic value is often the main reason for hosting sport mega-events (Wolfe et al., 2021). It was projected that hosting the Tokyo Olympics without spectators would result in a financial loss of up to 2.4 trillion yen in Japan (Blair, 2021). On the other hand, it was estimated that the Tokyo 2020 Olympic Games would positively impact Japan. Approximately 20 trillion Japanese yen was estimated for Tokyo alone as a city and 32 trillion Japanese yen for the whole of Japan (Japan Times, 2021). These figures indicate that the Japanese government expected the Games would bring huge growth across its society, economy and culture. Unfortunately, with no foreign visitors allowed and a rise of COVID-19 infections in Japan, this was not attainable. In addition, the outrage of Japanese citizens who took to the streets to protest the Games (Oi, 2021) adds to the case that COVID-19 is a hybrid threat.

So, we conceptualize COVID-19 as a hybrid threat to the Games because it is a biological and hybrid threat that became a global threat to all nations rather than one nation against another as suggested by NATO, so we argue, needed a different response to solving it. COVID-19 was only going to be solved by all nations working together against this hybrid threat to create a vaccine to survive and thrive. These values constitute the foundation on which the Olympic Movement builds its activities to promote sport, culture, and education to build a better world and reduce inequality (Kufenko and Geloso, 2021). Thus, COVID-19 as a hybrid threat to the world needed a coordinated approach to promote peace and harmony ensuring those nations that could not afford the vaccine were helped and not seen as a bidding war only for the developed nations who could afford it. Pressure from Nato, the UN, and others helped that process. If we look at initiatives such as Sport for Peace and Sport Development ethos of the Olympics, we also see that altruistic approach to helping developing nations. COVID-19, as a hybrid threat, threatened all that the Olympics stood for as a force for good. For example, the Games usually serve as a benefactor to other nations, athletes, and wider stakeholders and allows the host to give out promotional items to spectators at the opening ceremony that convey the image and branding that the hosts want to portray. At Tokyo 2020 the opportunity and potential benefit of this type of activity was lost. This brought questions of joint responsibility between the IOC and the Japanese government to the fore and issues of integrity, honesty and trust in each other to do the right thing and for Japan to lead the way with the IOC in demonstrating the values of Olympism in their response to tackling the hybrid threat. We, therefore, suggest that the unification of nations to combat COVID-19 as a hybrid threat emulates the values of Olympism, excellence, friendship and respect. This constituted the foundation of the Olympic movement, which is built on promoting sport, culture, and education with a view of helping to create a better world. Reflecting on the delivery of the Games suggests we need a different approach if we are to anticipate future virus or hybrid threats to the Games. A different model of delivery would help ease pressure on one Country or City to deliver the Games. We have started to see this with other events, countries bidding to host sports mega-events jointly, such as the Nordic Countries bidding to host the 2024 and 2028 European Football Championship. A model of sustainability, cost reduction, environmental reduction and venue maximization allows visitors to travel to different countries more locally and reduce the impact on one city or country. This we would suggest would reduce the spread of infection of future viruses with less people in one location and the economic and social benefits, and soft power benefits, being rewarded to more than one country with less environmental impact overall.

## Conclusion

This paper has taken a novel approach to conceptualize the effects of COVID-19 as having connected similarities with the term hybrid threat. The conflict of governments or political actors is a hybrid threat surrounding the discourse on the appropriate measure to combat pandemics. These conflicts are created within a constituency, which do not unify around a policy. The occurrence of global or national discomfort on the measures to combat COVID-19, including anti-government protests and forces in society willing to attack rather than engage in the policymaking process with violence or civil disobedience, highlights the similarities and connectedness between hybrid threats and COVID-19 pandemic. This similarity also includes the lack of trust in the government policymaking process, sapping a government's legitimacy. However, trust is a key indicator of a country's soft power, but the emergence of the COVID-19 developed a lack of trust in government policies. The nuances of tackling the virus are not straightforward, confirming our argument as to why the COVID-19 pandemic should be classified as a hybrid threat. The pandemic has caused the loss of jobs, freedom of movement and association. It has also led to the most significant global recession in history. All these effects are associated with conventional warfare or irregular warfare such as terrorism in a victimized country. We have argued that the definition of hybrid threat should expand beyond territorial and inter-state aggression.

In the case of the Tokyo 2020 Olympic Games as a vehicle to increase Japan's soft power; the Government based this desire on the evidence from the 1964 Olympic Games that served as a soft power tool for a re-acceptance of Japan into the global community as a peaceful nation. We connect the Tokyo 2020 Olympic Games to the political agenda of Japan's former Prime Minister Abe, who sought to strengthen Japan's position domestically, regionally and internationally as a first-tier country. The circumstances that the Games were organized behind closed doors to foreign visitors are disappointing for Japan's strategy to leverage the Games as a soft power tool. Hosting the Games behind closed doors signifies the significant impact of COVID-19 on Japan's foreign policy to use the Games as a soft power tool. Therefore, we sought in this paper to offer a novel approach to re-conceptualizing COVID-19 as a hybrid threat to sport mega-events but also to bring into question the basis for Governmental planning of the use of sports events in soft power strategies that is of international significance to policymakers and host nations. The lack of Plan B against the emergence of a biological threat leading from the pandemic, by the IOC and Tokyo Government is a worrying issue, that could easily be repeated if a unified response and plan is not created now to respond to future hybrid threats. We argue that a biological threat like COVID-19 is a hybrid threat and a complex problem that the IOC must seek to provide a plan for such eventualities in future even if it cannot action a solution on its own. Within the pandemic, the centralization of a global event has not proven to be effective both economically and socially in most cases. Hosting a sport mega-event like the Olympics in one city such as Tokyo resulted in questions being asked about the even distribution of wealth, legacy and sustainability of such event and adds to previous suggestions that events should be hosted in multiple cities at once (Greenwell, 2016). The emergence of COVID-19 revealed the lack of preparedness and exploited the vulnerability of social and economic institutions. However, we assert that there is a need to rethink and consider COVID-19 as a hybrid threat. We have offered a re-conceptualization of COVID-19 as a hybrid threat to all nations because it needed a different response and approach by a collective group of institutions, agencies and people. We, therefore, suggest that the unification of nations to combat COVID-19 as a hybrid threat emulates the values of Olympism, excellence, friendship and respect. This constituted the foundation of the Olympic movement, which is built on promoting sport, culture, and education with a view of helping to create a better world. Thus, COVID-19 as a hybrid threat to the Games, we suggest, became symbolic of the hybrid threat to the world. Just as nations united to ensure that those countries that could not afford the vaccine were helped, the Olympics had the chance to show they too could lead the way in their approach to ensuring athlete safety, visitor safety and citizens' safety. They had some success with that in that very few athletes missed the Games, and as a nation their infection rate was still lower than many other nations but COVID-19 as a hybrid threat, threatened all that the Olympic Games stood for, as a force for good. We finish by suggesting that a new approach to Games delivery is sought, one that unites countries to bid together, deliver together and spread the risk of future hybrid threats, whilst simultaneously doing the right thing. The right thing in soft power terms, but for the right reasons: the environment, sustainability, wellbeing, and development. Whether Japan's rankings in the soft power indices will be affected only time will tell, but this is not a problem for a country alone to address, the event owners of sports mega-events need to take more responsibility to work together to embrace an alternative model of delivery. Perhaps then, we may remind ourselves of the Olympic values that will help us tackle future hybrid threats.

## Author Contributions

SI was the lead author on this paper and led on the conceptualization of the hybrid threat of COVID-19. SI and GM contributed to the ideas, concepts, and writing of the paper. All authors contributed to the article and approved the submitted version.

## Conflict of Interest

The authors declare that the research was conducted in the absence of any commercial or financial relationships that could be construed as a potential conflict of interest.

## Publisher's Note

All claims expressed in this article are solely those of the authors and do not necessarily represent those of their affiliated organizations, or those of the publisher, the editors and the reviewers. Any product that may be evaluated in this article, or claim that may be made by its manufacturer, is not guaranteed or endorsed by the publisher.
